# Monosynaptic targets of utricular afferents in the larval zebrafish

**DOI:** 10.3389/fneur.2022.937054

**Published:** 2022-07-22

**Authors:** Yizhen Jia, Martha W. Bagnall

**Affiliations:** Department of Neuroscience, Washington University, St. Louis, MO, United States

**Keywords:** vestibular, balance, utricle, electron microscopy, zebrafish

## Abstract

The larval zebrafish acquires a repertoire of vestibular-driven behaviors that aid survival early in development. These behaviors rely mostly on the utricular otolith, which senses inertial (tilt and translational) head movements. We previously characterized the known central brainstem targets of utricular afferents using serial-section electron microscopy of a larval zebrafish brain. Here we describe the rest of the central targets of utricular afferents, focusing on the neurons whose identities are less certain in our dataset. We find that central neurons with commissural projections have a wide range of predicted directional tuning, just as in other vertebrates. In addition, somata of central neurons with inferred responses to contralateral tilt are located more laterally than those with inferred responses to ipsilateral tilt. Many dorsally located central utricular neurons are unipolar, with an ipsilateral dendritic ramification and commissurally projecting axon emerging from a shared process. Ventrally located central utricular neurons tended to receive otolith afferent synaptic input at a shorter distance from the soma than in dorsally located neurons. Finally, we observe an unexpected synaptic target of utricular afferents: afferents from the medial (horizontal) semicircular canal. Collectively, these data provide a better picture of the gravity-sensing circuit. Furthermore, we suggest that vestibular circuits important for survival behaviors develop first, followed by the circuits that refine these behaviors.

## Introduction

The vestibular system is vital for coordinated movement in vertebrates. Vestibular end-organs and neuronal circuits develop early, *in utero* for mammals. As a consequence, it has been challenging to study the organization and development of vestibular circuits (1, 2). Significant open questions include the sequence of developing synaptic connectivity, the molecular cues that specify appropriately directed reflex arcs, and the role of activity-dependent plasticity in circuit formation (2).

The limited accessibility of mammalian models for questions of early development can be mitigated by use of other vertebrates with external development, including frogs and fishes. At just 3 days post fertilization (dpf), larval zebrafish hatch from the chorion and begin to exhibit basic vestibular-dependent functions, including the vestibulo-ocular reflex (VOR) and orientation with respect to gravity (2–5). These behaviors further refine over the course of development (6–8). Thus, the larval zebrafish provides a snapshot of circuitry at a period in which the vestibular reflex circuit has crucial elemental functions that will be subject to later maturation.

Even at these larval stages (5–7 dpf), vestibular signals are widespread throughout the brain, as assessed by calcium imaging with natural or artificial vestibular stimuli (9, 10). These signals are mostly or entirely dependent on pathways arising from the utricular otolith, because the semicircular canals are too small to be responsive to normal head movements at these ages, and the saccular otolith is specific for auditory responses (3, 8, 11–13). Indeed, selective activation of the utricle *via* optical trapping is sufficient to drive vestibular responses (14). In contrast, the saccule is more effective at transmitting acoustic information, possibly due to its larger size, as loss of the saccule but not the utricle dramatically increases hearing thresholds (15, 16). Therefore, the utricular afferents provide the sole source of vestibular information about head movement and orientation with respect to gravity at larval stages. However, there may be some overlap of auditory and vestibular signals, particularly in central pathways (17).

**Figure 1:**
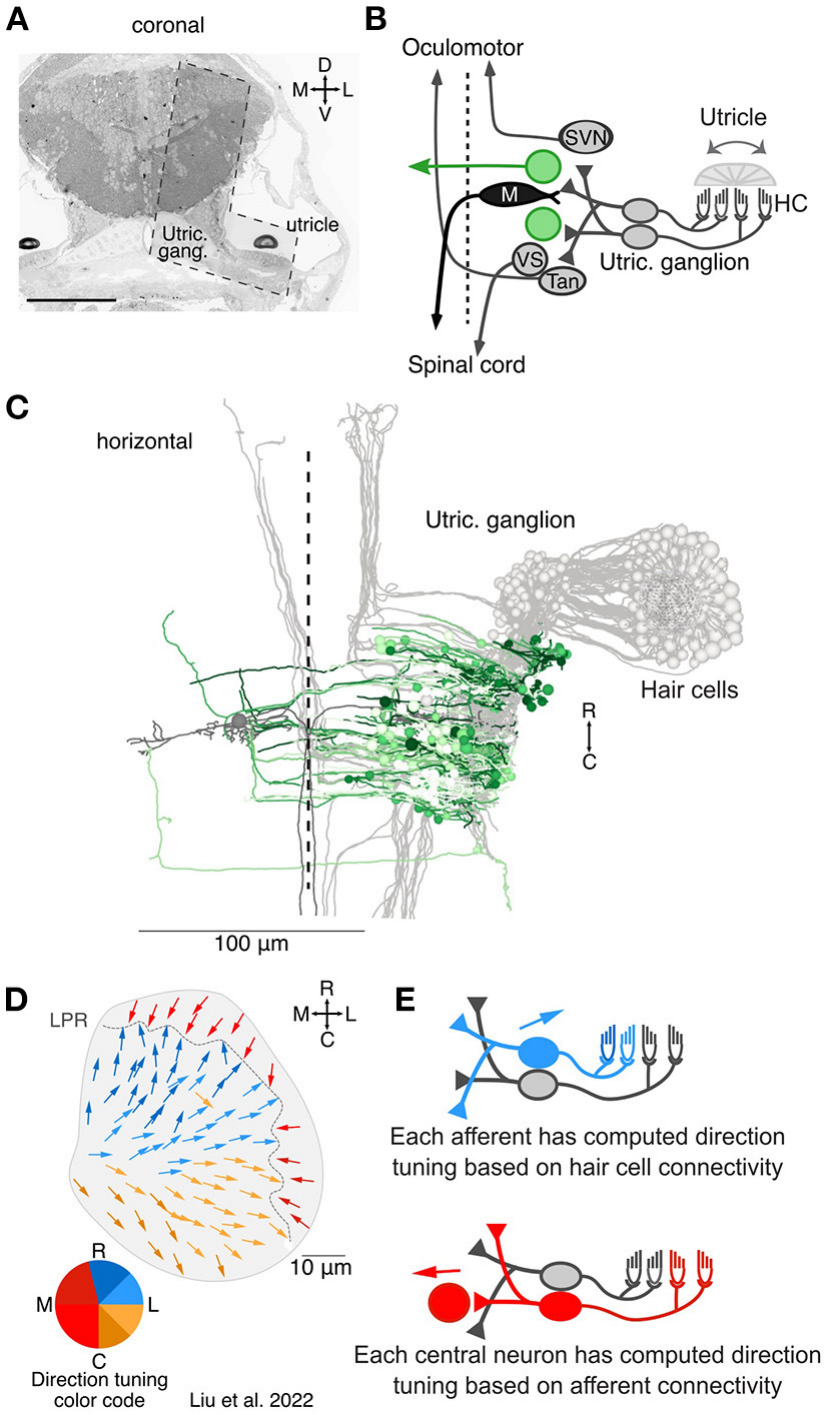
Overview of the central targets of utricular afferents. **(A)** Coronal section of 5.5 dpf larval zebrafish showing the total area imaged originally by Hildebrand et al. (18) and the area re-imaged at 4 × 4 × 60 nm^3^ per voxel (L shaped, dashed outline). This area was re-imaged across 105 μm in the rostrocaudal axis (1,757 coronal sections). Scale bar, 100 μm. Reprinted from (19). **(B)** Circuit schematic highlighting the central vestibular targets described here (green) in the context of the utricular / motor circuit (gray). We identified both commissurally-projecting central neurons and neurons whose axon could not be traced extensively. **(C)** Horizontal projection of the utricular circuit. As in **(B)**, greens represent neurons described here and grays represent neurons described in Liu et al. (19). Dashed line represents midline. Utricular afferent somata are in the utricular ganglion. **(D)** Directional tuning of all 91 hair cells in the utricular macula, horizontal view. Each vector represents the best direction of tilt responsiveness, as inferred from the positions of the kinocilium and stereocilia. To facilitate visualization, the directional tuning is also encoded with a color scheme, lower left. Tuning for ipsilateral and rostral tilt is shown in blue, for ipsilateral and caudal tilt in yellow, and contralateral tilt in red. The directional representation is slightly asymmetric to accommodate the patterns of the hair cells. LPR, line of polarity reversal. Data from Liu et al. (19). **(E)** Schematic of computed tuning for afferents and central neurons. Afferent tuning was computed as a weighted circular average based on the number of ribbon synaptic inputs from hair cells. Central neuron tuning was in turn computed as a weighted circular average based on the number of afferent synaptic contacts. We note that these directional tuning responses only take into account monosynaptic, utricular inputs, not canal inputs or polysynaptic pathways.

The arrival of large-scale serial-section electron microscopy in larval zebrafish (18) has provided an opportunity for analysis of the utricular circuit. Recently, we reconstructed the vestibular hindbrain at synaptic resolution, allowing us to visualize connectivity of utricular afferents from their peripheral hair cell inputs all the way to central targets, including the Mauthner cell and the tangential, vestibulospinal, and superior vestibular nuclei (Figures 1A,B) (19). Here we expand that analysis to reveal the other targets of direct utricle afferent input, including commissural neurons and several neurons with unknown identities. We define the inferred directional tuning of these neurons to show that neurons carrying contralateral head tilt information are predominantly found at the lateral margin of the vestibular hindbrain at this stage in development (5.5 dpf). In addition, we find surprising axo-axonic synaptic connections from utricular afferents onto a few medial (horizontal) semicircular canal afferents, suggesting a possible developmental mechanism for coordinating signals from these two independent sensors of head movement.

## Methods

The serial-section EM database was acquired as previously described (19, 20) from the right side of one larval zebrafish (18). In brief, an ultrathin (60 nm) section library was previously generated from the head and rostral spinal cord of a single 5.5 dpf larval zebrafish (18). These sections were originally imaged at lower resolution (either 18.8 × 18.8 × 60 or 56.4 × 56.4 × 60 nm^3^/voxel) to cover the entire brain. Thanks to a generous loan of a subset of sections from the Engert lab, our lab re-imaged the right side of the fish, covering the utricular hair cells, utricular afferents (identified by their peripheral processes reaching the utricular macula), and a rostrocaudal extent of the brainstem that covered several major vestibular nuclei at 4.0 × 4.0 × 60 nm^3^/voxel using a Zeiss Merlin 540 FE-SEM with a solid-state backscatter detector. These sections were aligned onto the original dataset using the TrakEM2 plugin in FIJI (21, 22), with custom support from UniDesign Solutions. The resulting image dataset is hosted and publicly accessible at http://zebrafish.link/hildebrand16/data/vestibular_right which includes access to all connectivity data. Additional access for further reconstructions can be obtained by contacting the authors.

Utricular hair cells were identified by location in the utricular macula and ciliary projections. For each hair cell, kinocilium and stereocilia positions were marked at the apical surface, and inferred tuning direction was calculated by creating a vector from the center of mass of stereocilia to the kinocilium (19).

Using CATMAID for skeleton reconstructions (23), each utricular afferent was identified by following each postsynaptic contact with a utricular hair cell ribbon synapse. In total we identified 944 ribbon synapses, of which only 1.6% (15/944) postsynaptic processes could not be followed due to the quality or ambiguity of the images. Therefore, the 105 utricular afferents identified here likely represent close to the complete set of utricular afferents that could be functional at this stage of development, with perhaps 2–3 missing. However, additional afferents are likely added later in development. Centrally, each utricular afferent was followed throughout its central process to identify all synaptic connections in the brain. Each postsynaptic contact of utricular afferents was reconstructed as far as possible. If the associated soma was found, we then reconstructed every process emerging from that soma as completely as possible. Only neurons in which a soma was identified are described here, as the remaining fragmentary reconstructions are not suitable for interpretation.

Each afferent was assigned an inferred utricular tuning as a weighted average of the hair cells contacting it (average ± standard deviation, 3.0 ± 1.5 hair cells contacting each afferent). Each central neuron was in turn assigned an inferred utricular tuning as a weighted average of the afferents contacting it. All tuning is expressed in the reference frame of animal tilt, with rostral tilt equivalent to nose-down pitch; caudal tilt to nose-up pitch; ipsilateral tilt to rightward roll; contralateral tilt to leftward roll.

Neuron position was calculated from the three-dimensional coordinates of the center of each soma, as marked in CATMAID. Distance along neurites was calculated by summing the Euclidean distances between each node in the skeletonized reconstructions. Analyses and statistics were carried out in Igor Pro 6 (Wavemetrics), with specific tests as stated in the text.

## Results

To form a picture of all the brainstem neurons receiving direct monosynaptic input from the utricular nerve, we first traced utricular afferents, identified by their postsynaptic contacts with utricular hair cell ribbon synapses, on the right side of the brain in one 5.5 dpf larval zebrafish (18, 19). We identified 105 utricular afferents, which likely represent close to a complete count of functional afferents at this developmental stage, as only 1.6% (15/944) utricular ribbon synapse postsynaptic contacts could not be followed back to the the utricular ganglion. These afferents were reconstructed throughout their central projections in the brainstem, bounded caudally by the tangential nucleus and rostrally by the superior vestibular nucleus.

At every synaptic contact from a utricular afferent onto a central neuron, as identified by a darkened cleft and presynaptic vesicles, we reconstructed the postsynaptic process as far as possible. Out of 2,075 total identified synaptic connections, 1,311 postsynaptic processes (63%) were reconstructed all the way back to a total of 203 brainstem somata that are targets of utricular afferent input. The remaining 764 postsynaptic processes could not be followed all the way to a soma because of uncertain continuations between sections, and therefore were not analyzed further. From the soma, all neurons were reconstructed as far as possible. Limitations on reconstruction typically arose from either thin processes with uncertain continuations or where processes exited the volume that had been re-imaged at high resolution. In about half of the central target somata (108 of 203), the axon could be identified by characteristic appearance; in some cases, myelination permitted reconstruction beyond the bounds of the re-imaged volume.

Of the 203 somata of neurons receiving direct utricular afferent input, 43 were identifiable by position and axon trajectory as belonging to the Mauthner cell or the tangential, superior vestibular, or vestibulospinal nuclei; these were characterized in a recent publication (gray and black neurons, Figures 1B,C) (19). Here we characterize the anatomy and inferred directional tuning of the remaining 160 neurons, whose identity is less certain (green neurons, Figures 1B,C). We had previously reconstructed each utricular hair cell and derived its directional tuning as a vector from the center of mass of the stereocilia to the kinocilium (all 91 hair cells; Figure 1D). The tuning of each utricular afferent was calculated by averaging its hair cell inputs, weighted by the number of synaptic ribbons at each connection. In turn, the tuning of each central brainstem neuron was calculated as a weighted average of its afferent vector inputs (Figure 1E). We note, however, that this inferred tuning does not include any potential inputs from the semicircular canals, contralateral vestibular pathways, or other sources. Therefore, the directional tuning presented here is solely what is predicted from monosynaptic utricular afferent input.

As shown in Figure 1D, the computed directional tuning is represented with a color code to facilitate visualization, both here and through the remaining figures. Blue colors represent best responses to head tilt in the ipsilateral, rostral direction (pitch nose-down and rightward roll). Yellow colors represent best responses to head tilt in the ipsilateral, caudal direction (pitch nose-up and rightward roll). Red colors represent best responses to head tilt in the contralateral direction (leftward roll).

**Figure 2:**
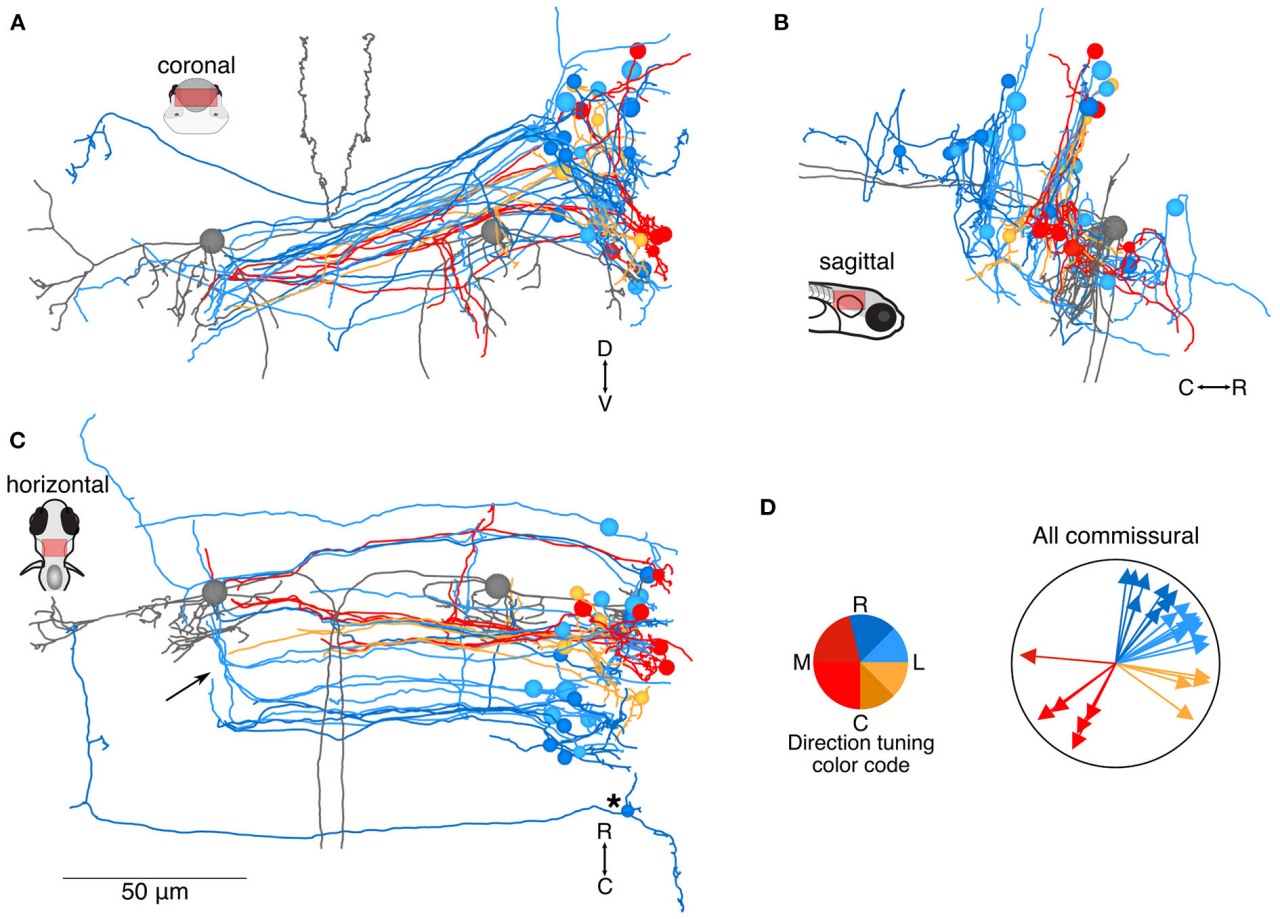
Location and directional tuning of identified commissural neurons receiving utricular input. **(A)** Coronal projection of reconstructions of 31 commissural neurons that receive direct utricular afferent input, colorized by their inferred directional tuning [see color code, **(D)**]. In addition, the left and right Mauthner cells are shown in gray to facilitate comparisons with other results. Note that the Mauthner cells are shown as spheres. Nearly all commissural utricular neurons follow a similar axon projection path. **(B)** Sagittal view of the same neuron reconstructions as in **(A)**. Most of these commissural neurons are located caudally to the Mauthner cell bodies. **(C)** Horizontal view of the same neurons as in **(A)**. Arrow points to a position where several commissural utricular axons can be seen to turn rostrally, in the same mediolateral position as the Mauthner cell body. Asterisk indicates the presumed tangential neuron described in text. Scale bar applies to **(A–C)**. **(D)** Summary of the inferred directional tuning of commissural utricular neurons. Vector direction indicates the computed directional tuning of each neuron. Arrow lengths have been adjusted to facilitate visualization.

The vestibular brainstem contains strong commissural connections, both excitatory and inhibitory, in frogs, mice, and primates (24–29). We found that 19% (31/160) central neurons of our reconstructed population projected axons commissurally. These axons typically followed a slope from dorsal to ventral as they crossed the midline (coronal view, Figure 2A; Mauthner cells are included in gray for context), similar to the arcuate fiber tracts seen in frog commissural vestibular neurons (28) and possibly the inhibitory Gsx1+ population identified in zebrafish (30). There was some variation in the dorsoventral elevation, as visible in the sagittal view (Figure 2B). Commissural neuron soma distribution in the rostrocaudal axis was bimodal, with a smaller population rostrally, few neurons at the level of the Mauthner cells, and a larger population caudal to the Mauthner cell (horizontal view, Figure 2C), similar to the reported distribution in frogs (28). The direction tuning of these neurons varied as indicated by their color. Both ipsilateral (blue, yellow) and contralateral (red) tilt information is carried across the midline, although in different proportions in this dataset: 74% ipsilateral tilt (23/31) and 26% contralateral tilt (8/31) (summary, Figure 2D). Axons from a subset of commissural neurons could be followed through a rostral turn, midway across the contralateral brainstem, near the Mauthner soma; all of these neurons had inferred tuning for rostral ipsilateral tilt (arrow, Figure 2C). In addition, at least four neurons appeared to make contact with the contralateral Mauthner cell and exhibited inferred contralateral tilt tuning (red) (19). Finally, one neuron (asterisk) projected a very long axon that reached the position of the predicted contralateral vestibular nuclei, reminiscent of the tangential commissural neuron anatomy described by Bianco and colleagues (6). This and other putative tangential nucleus neurons are also described in **Figure 5**.

**Figure 3:**
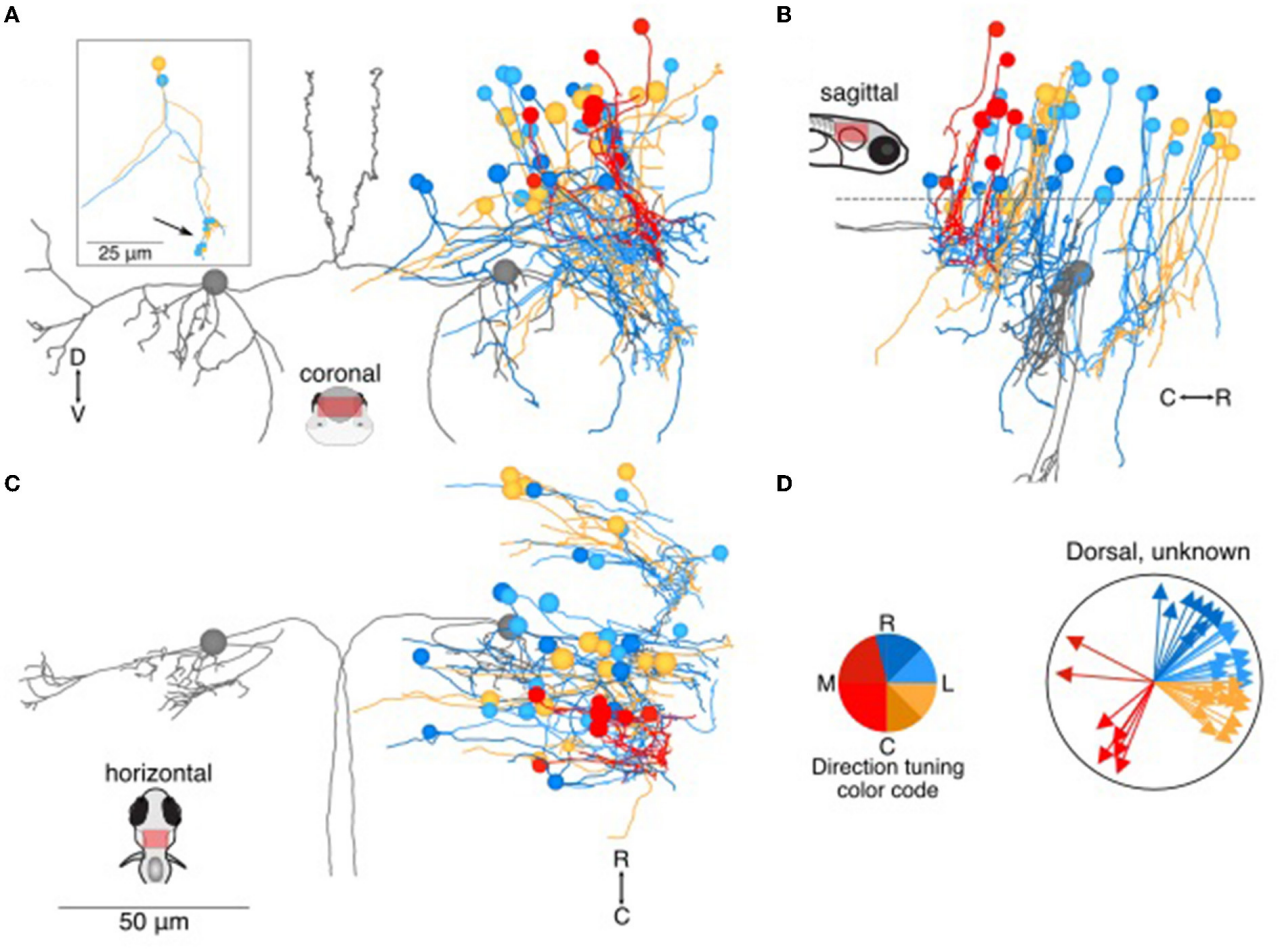
Location and directional tuning of dorsally located neurons receiving utricular input. **(A)** Coronal projection of reconstructions of 46 dorsal neurons (>20 μm dorsal to Mauthner cell) that receive direct utricular afferent input, colorized by their inferred directional tuning [see color code, **(D)**]. In addition, the left and right Mauthner cells are shown in gray to facilitate comparisons with other results. Inset, two example neurons with the locations of their synaptic inputs (arrow, small circles). The processes extending to the left of the reconstruction are presumed axons. **(B)** Sagittal view of the same neuron reconstructions as in **(A)**. Dashed line indicates the plane separating dorsal neurons from ventral neurons in Figure 4. **(C)** Horizontal view of the same neurons as in **(A)**. Scale bar applies to **(A–C)**. **(D)** Summary of the inferred directional tuning of dorsal neurons with utricular input. Vector direction indicates the computed directional tuning of each neuron. Arrow lengths have been adjusted to facilitate visualization.

Of the remaining 129 neuron somata, we either could not identify the axonal process or could not reconstruct it over long distances. Therefore, it is difficult to assess anatomical identity. To facilitate description of these neurons, we have divided them into a dorsal and ventral category based on the soma position, with the dividing line ~20 μm dorsal to the Mauthner cell body. Dorsally located utricular target neurons (*N* = 46, Figure 3A) typically extended long dendrites ventrally, where they received synaptic inputs from the utricular afferents. Several of these dorsal neurons shared a general morphology with identified commissural neurons: a unipolar soma extending a long neuronal process that splits into a commissurally-directed axon and an ipsilaterally-directed dendrite (inset, Figure 3A). Of the identified commissural neurons (Figure 2), 7/15 dorsally located neurons were unipolar, in contrast with 0/16 ventrally located neurons. This unipolar morphology is common in zebrafish as well as invertebrates, but to the best of our knowledge rare in the mammalian vestibular system. Somata were located as far as ~50 μm dorsal to the Mauthner cell (Figure 3B). Based on the dorsoventral age relationship in zebrafish hindbrain (31), dorsally located neurons are likely to be younger, and therefore the unipolar morphology may be transient. Similar numbers of neurons exhibited inferred tuning to rostral and caudal ipsilateral tilt, with a smaller, mostly caudally positioned subset exhibiting inferred tuning to contralateral tilt (Figures 3C,D).

**Figure 4:**
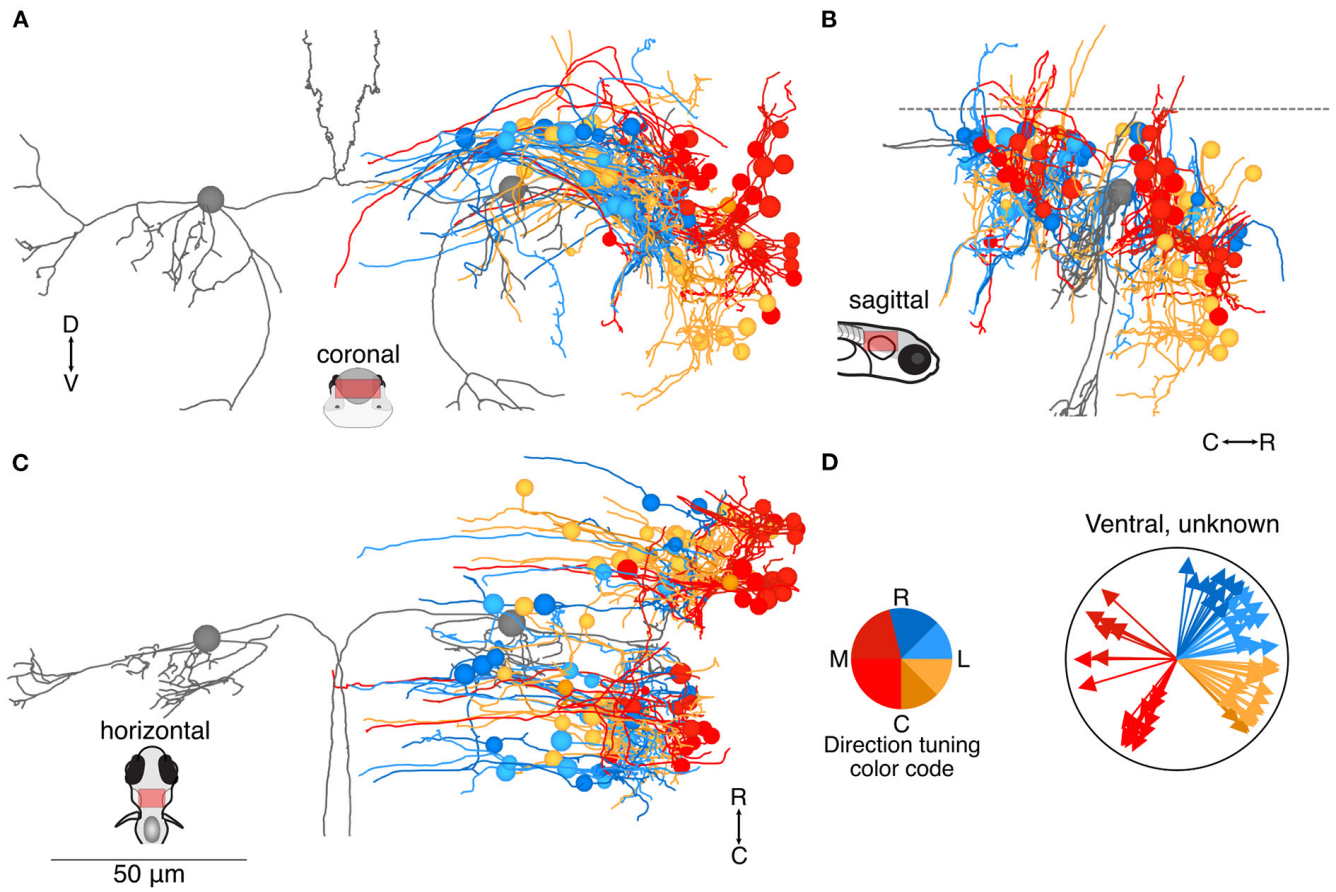
Location and directional tuning of ventrally located neurons receiving utricular input. **(A)** Coronal projection of reconstructions of 83 ventral neurons that receive direct utricular afferent input, colorized by their inferred directional tuning [see color code, **(D)**]. In addition, the left and right Mauthner cells are shown in gray to facilitate comparisons with other results. Neurons responsive to contralateral tilt (reds) are preferentially located on the lateral edge of the vestibular brainstem (see Figure 7). **(B)** Sagittal view of the same neuron reconstructions as in **(A)**. Ventrally located neurons are distributed both rostrally and caudally to the Mauthner cell body. **(C)** Horizontal view of the same neurons as in **(A)**. Scale bar applies to **(A–C)**. **(D)** Summary of the inferred directional tuning of ventral neurons with utricular input. Vector direction indicates the computed directional tuning of each neuron. Arrow lengths have been adjusted to facilitate visualization.

Another 83 neuron somata were located more ventrally (Figure 4), and are presumably earlier-born (31). In this group, neurons with inferred tuning to contralateral tilt were located noticeably more laterally than neurons with inferred tuning to ipsilateral tilt (Figure 4A). The ventral neurons were roughly equally divided between rostral and caudal to the Mauthner cell (Figures 4B,C). As in the dorsal group, many of these neurons appeared to extend initial axons toward the midline (Figure 4C), although the eventual destination was unknown. The pool of ventrally positioned unidentified neurons was roughly evenly split in their inferred directional tuning (Figure 4D).

**Figure 5:**
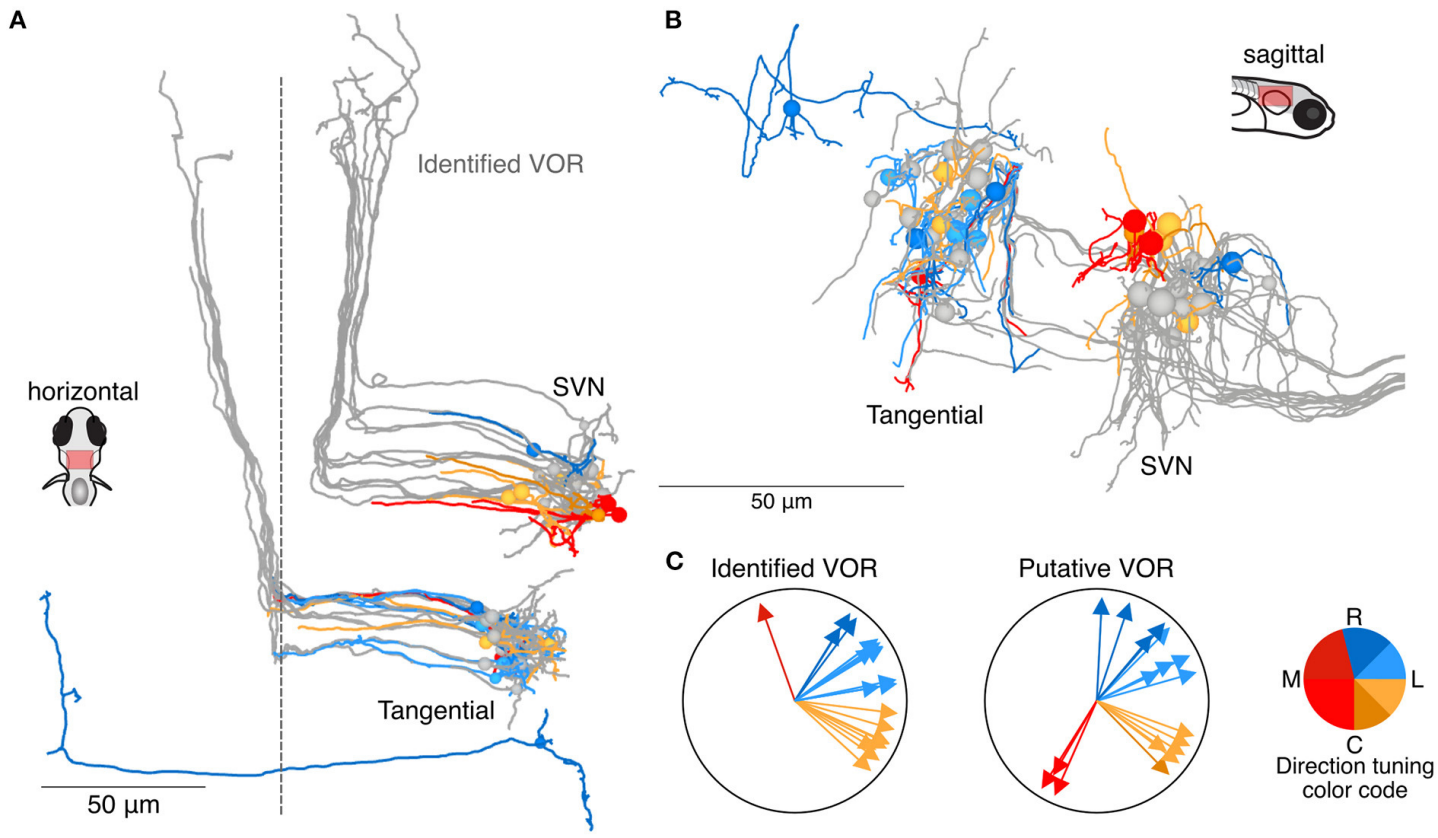
Utricular-recipient neurons putatively involved in the VOR. **(A)** Horizontal projection of reconstructions of 11 identified tangential nucleus and 12 identified superior vestibular nucleus neurons (grays) along with 11 putative tangential and 6 putative SVN neurons that receive direct utricular input (colors). Neurons were identified as putative VOR neurons based on soma position and initial axon trajectory. One putative tangential nucleus neuron (dark blue, caudally located) was identified based on apparent homology to commissural neurons described by Bianco et al. **(B)** Sagittal view of the same neuron reconstructions as in **(A)**. **(C)** Summary of the inferred directional tuning of identified (left) and putative (right) VOR neurons with utricular input.

Among the 129 neurons whose axons could either not be reconstructed over long distances or not identified with certainty (Figures 3, 4), we identified 18 neurons as putative members of the tangential or superior vestibular nuclei, based on their anatomical position and initial axon trajectory. Neurons with a soma and axon that appeared qualitatively similar to identified tangential or superior vestibular nucleus (SVN) neurons are shown in Figure 5 (in color) along with the previously reconstructed tangential and superior vestibular nucleus neurons (in gray) identified as part of the VOR pathway. Though we have only moderate confidence in their identity, we include these neurons to show that these nuclei are likely to contain more neurons than we were able to reconstruct with confidence. We note that an additional set of identified tangential and SVN neurons received no utricular input but some input from the anterior or posterior canals (data not shown), and therefore would be selective for rotational but not inertial stimuli (D. Goldblatt and D. Schoppik, personal communication). No additional putative members of the vestibulospinal population have been identified, but some likely exist based on the gap between our 19 identified vestibulospinal neurons and a reported average of 27 (32).

**Figure 6:**
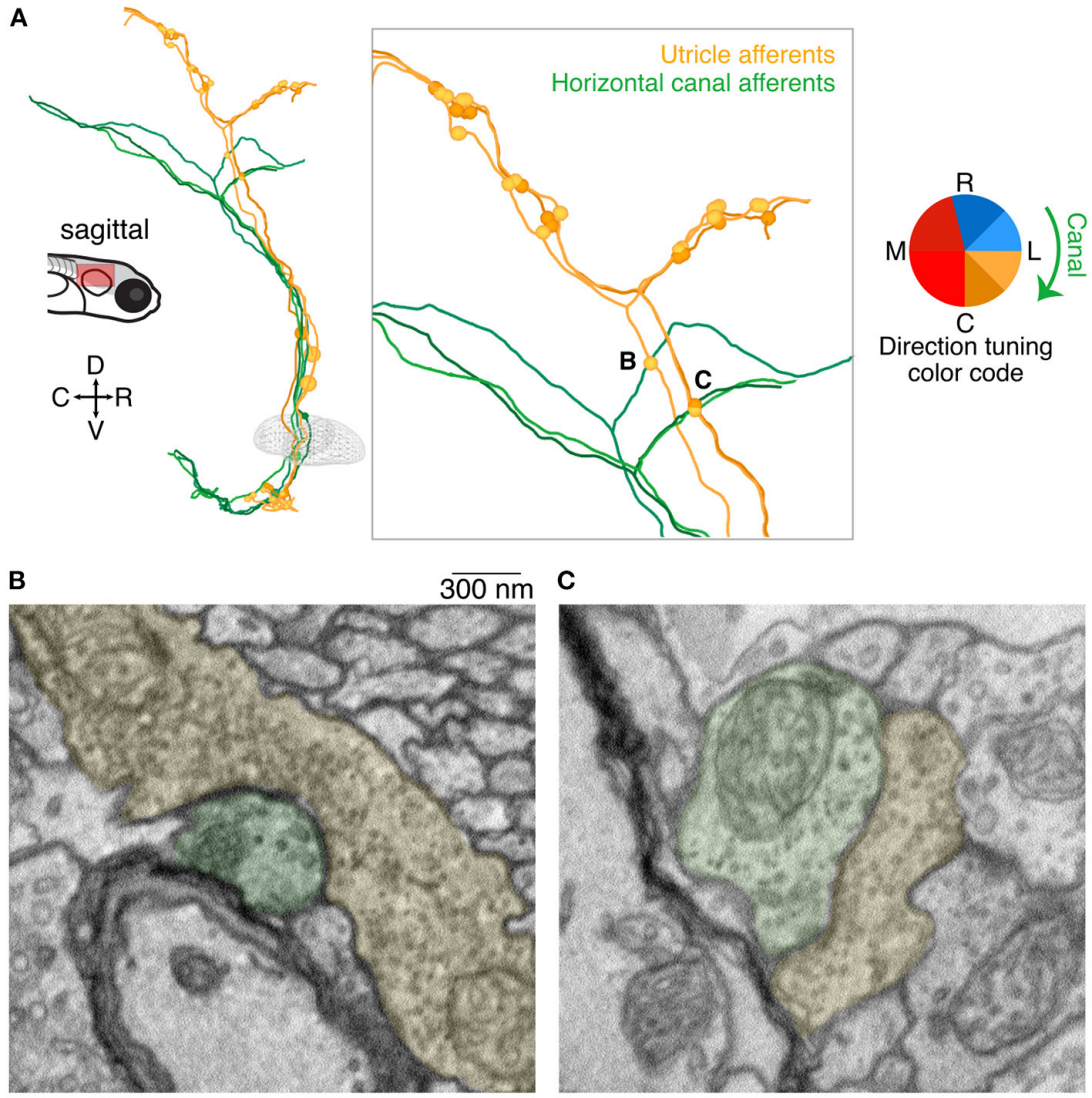
Axo-axonic synaptic connections from utricular afferents to horizontal canal afferents. **(A)** Sagittal projection of reconstructions of three utricular afferents (yellows) and three horizontal canal afferents (greens). Each utricular afferent makes a synaptic connection onto one canal afferent, shown as small circles in the inset panel. Notably all of these apparent connections were found in the ascending branch of the utricular afferent before its central bifurcation, where no other synaptic connections were identified. Inset letters indicate the two synaptic connections shown in panels B and C. **(B)** Electron micrograph of one of the axo-axonic synapses shown in **(A)**. The utricular afferent (pseudocolored yellow) appears to have multiple release sites onto the canal afferent (pseudocolored green). Scale bar applies to both B and C. **(C)** As in **(B)**, for a second connection. The postsynaptic density is less pronounced in this image, but nonetheless the clustered vesicles suggest a synaptic connection.

In addition to the central targets of utricular afferents described above, we found another surprising population that appears to receive utricular afferent input: axonal afferents from the medial semicircular canal. Canal afferents have been only partially reconstructed in this dataset, as our scope is limited to those afferents that were sufficiently myelinated to allow reconstruction outside of the imaged volume (18). Nonetheless, we found likely synaptic connections from three utricular afferents onto three medial (horizontal) canal afferents (Figure 6A). The utricular and canal afferents travel in similar courses as they exit the vestibular ganglia, but then diverge prior to bifurcation (Figure 6A). However, the ascending branch of each of these utricular afferents, prior to bifurcation, formed axo-axonic contacts onto the rostrally directed branch of a medial canal afferent (inset, Figure 6A). Examples of these synaptic contacts are shown in Figures 6B,C. In Figure 6B, multiple putative release sites are visible, suggesting a particularly strong connection. All three utricular afferents had inferred tuning to caudal ipsilateral tilt (yellows). Although we did not characterize the hair cells of the medial canal, it is well-established that these afferents should carry signals for ipsiversive head rotation (green clockwise arrow, Figure 6A). It is unclear how widespread this phenomenon is, as we found only three synaptic connections, but the consistent spatial relationship of these connections is striking.

**Figure 7:**
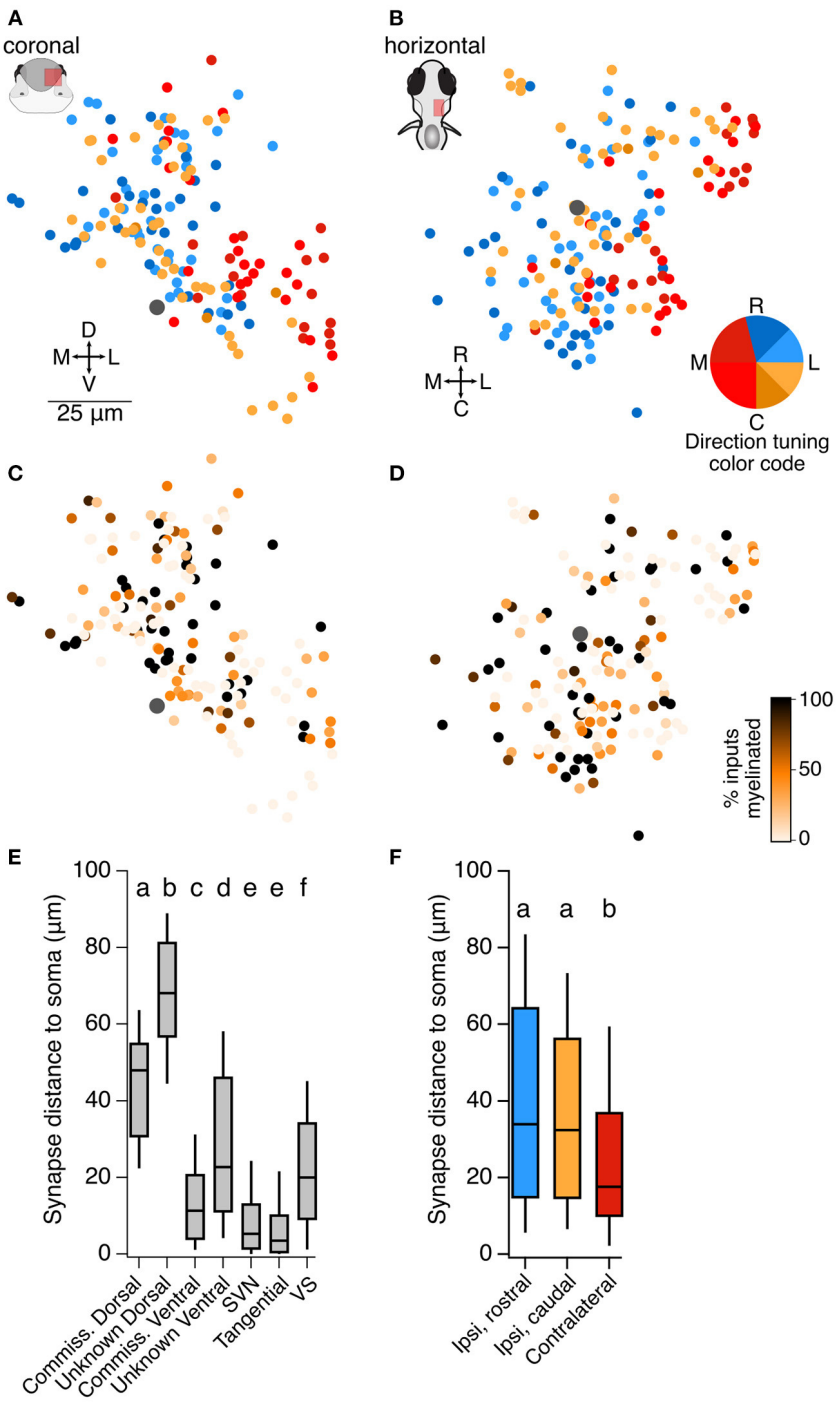
Organization of central utricular neurons by inferred directional tuning. **(A)** Summary of the inferred directional tuning of all neurons described here in the coronal plane. The Mauthner cell body is indicated by a large gray dot. Neurons predicted to respond to contralateral tilt (reds) are on average located more laterally than those responsive to ipsilateral tilt, Wilcoxon-Mann-Whitney test, *p* = 1.0 × 10^−9^. **(B)** As in **(A)**, in the horizontal plane. Neurons predicted to respond to contralateral tilt are on average located more ventrally, Wilcoxon-Mann-Whitney test, *p* = 0.007. **(C)** Summary of the fraction of inputs that arise from myelinated vs. unmyelinated utricular afferents for all neurons described here. Darker colors represent more input from myelinated afferents (scale in **D**). Mauthner cell is indicated by a large gray dot. There is no apparent spatial organization of neurons receiving more or less myelinated input, although central neurons with myelinated axons receive more input from myelinated afferents (see Discussion). **(D)** As in **(C)**, in the horizontal plane. **(E)** The average distance of utricular synaptic contacts from the soma, measured along the dendrite, for each of the classes of neurons described here. Dorsally located somata receive utricular synaptic input at a longer distance from the soma than ventrally located somata of the same class (compare commissural dorsal and ventral neurons, and unknown dorsal and ventral neurons). Box plot displays median and 25–75th % iles; whiskers are 10–90th % iles. Small lettering above bars represents statistical relationships. Although most groups are significantly different from each other, the differences in values are modest in most cases, with the exception of the dorsal and ventral populations. Pairwise comparisons for these groups: Wilcoxon-Mann-Whitney between commissural dorsal and commissural ventral (Figure 2), *p* = 4 × 10^−10^; between unknown dorsal and unknown ventral (Figures 3, 4), *p* = 0. **(F)** The average distance of utricular synaptic contacts from the soma, measured along the dendrite, for central neurons separated by their inferred utricular tuning (not including SVN, tangential, and VS neurons). Neurons with inferred contralateral tilt sensitivity (red) received input significantly closer to the soma than neurons with inferred ipsilateral tilt sensitivity (blue, yellow). Small lettering above bars represents statistical relationships. Kruskal-Wallis test among all three groups, *p* = 0.005. Wilcoxon-Mann-Whitney tests between ipsi-rostral and ipsi-caudal groups, *p* = 0.35; between ipsi-rostral and contralateral, *p* = 0.002; between ipsi-caudal and contralateral, *p* = 0.005.

At the level of overall topography of the vestibular brainstem, we find that neurons receiving monosynaptic information about contralateral head tilt are located on average 25 μm more laterally and 8.5 μm more ventrally than neurons receiving monosynaptic information about ipsilateral head tilt. This is seen in summary plots of soma position for all neurons described in Figures 2–4, 7A,B. In contrast, although central neurons varied widely in the fraction of their input that comes from myelinated vs. unmyelinated afferents at this stage, we did not detect any pattern in their distribution (Figures 7C,D). Dorsally located somata typically received otolith afferent synaptic input at over twice the distance from the soma than neurons in other positions (typically 45–65 μm from the soma, as compared to 10–20 μm from the soma for other populations; Figure 7E). However, neurons predicted to be responsive to contralateral tilt were usually located more ventrally (Figure 7A), close to the location of the otolith afferent arborizations, and accordingly they received synaptic input closer to the soma on average than neurons predicted to be responsive to ipsilateral tilt (average distance of otolith afferent synaptic input from the soma in ipsilateral rostral tilt neurons: 41 μm; ipsilateral caudal tilt neurons: 36 μm; contralateral tilt neurons: 24 μm; Figure 7F). We conclude that some directional topography exists in a fashion that could support head movement computations.

## Discussion

### Patterns of sensory encoding

Many sensory systems, including visual, auditory, and somatosensory, exhibit well-defined topographical organization with respect to tuning features. In contrast, the vestibular brainstem is largely organized around motor principles, with nuclei composed of neurons projecting to particular motor regions. Though we have recently demonstrated that the utricular afferent ganglion is organized rostrocaudally in a similar fashion to the hair cells, with afferents encoding rostral tilt located rostrally and afferents encoding caudal tilt located caudally (19), the central projections are not ordered with similar clarity. However, our data here do show that afferents encoding contralateral head tilt tend to contact central neurons located at the ventrolateral edge of the vestibular brainstem (Figures 4, 7). This pattern may be lost in later development, but it aligns with the observed afferent projection patterns (19) and suggests some relatively simple potential means of organizing direction-opponent circuits (33, 34). While we did not observe direct utricular input to the cerebellum at this stage, possibly because it extended outside the reimaged volume, our data do not support the finding in mouse that afferents innervating the lateral utricle, carrying contralateral head tilt information, are exclusively directed toward the cerebellum (35).

Two groups have reported whole-brain calcium imaging responses to vestibular stimulation in larval zebrafish (9, 10). The scope of our dataset is much more limited, to neurons receiving monosynaptic utricular afferent inputs within the unilateral vestibular hindbrain. Nonetheless, we observe some shared features of our results. First, many neurons encoding contralateral head tilt are located more laterally and ventrally to those encoding ipsilateral head tilt (Figures 7A,B), consistent with the more laterally displaced axons of contralateral tilt afferents. These results align with observed roll-tilt sensitivity in the vicinity of the vestibular nuclei in Migault et al. (their Figure 4F) (10). Favre-Bulle and colleagues found a more modest but putatively similar set of contralateral-roll responsive neurons at the lateral and ventral margins of the vestibular nuclei (their Figure 5I) (9). Interestingly, both labs reported hindbrain vestibular-responsive neurons, often with phase-shifted activity, located closer to the midline than any neurons identified in our dataset. We suggest that these neurons must receive indirect utricular afferent input, rather than monosynaptic, because we did not reconstruct any monosynaptic targets located that far medially. One candidate source for polysynaptic utricular input is commissural neurons with axonal arborization near the midline (Figure 2C, arrows). Alternatively, some of the target neurons we were not able to reconstruct may have somata this close to the midline.

It is unclear whether the dorsally located neurons (Figures 2, 3) are likely to exhibit significant somatic calcium responses to vestibular stimuli. Most of these neurons received relatively few utricular inputs [medians (25–75 percentiles): 4 [2–7] utricular inputs from 3 (2–4.75) discrete afferents] and those inputs are located a long distance from the soma (Figure 7E). This anatomical result may explain why this population does not appear strongly in the calcium imaging analyses, although it is hard to compare directly (9, 10). Indeed, the identities of these populations are obscure, and their function may be dominated by non-utricular inputs. The general motif of unipolar neurons with ipsilateral dendrites and commissural axons has also recently been described in GABAergic neurons of rhombomere 1 in zebrafish (36), suggesting perhaps a common morphological template.

### Axo-axonic connections among afferents

The process by which vestibular afferents target the correct type of central neuron is not yet known. Afferents from both the otoliths and the semicircular canals converge on many central neurons in frogs and primates (37, 38). However, while the directional tuning of all afferents arising from a given canal is uniform, different otolith afferents carry diversely tuned signals. Therefore, the development of tuning in central vestibular neurons, where convergent afferents from canals and otoliths must have similar tuning to drive aligned oculomotor responses (38–40), is a significant puzzle. One possibility is that central neurons carry a molecular identity that drives retrograde signals to instruct synaptic connectivity from afferents for, e.g., rostral vs. caudal tilt (41). A second possibility is that canal afferents, due to their uniform response directions, initially set up tuning in central vestibular neurons, which then selectively stabilize connections from appropriately tuned otolith afferents. This idea has received support from the observation that more directionally selective responses to translational stimuli arise in extraocular muscles over development, in a canal-dependent process (42), although it is unclear whether this refinement occurs at the afferent synapse onto vestibular nucleus neurons or the vestibular synapse onto oculomotor neurons. Here we present evidence for another possible source of coordination between canal and otolith afferents: axo-axonal synaptic connections. We found synaptic contacts specifically from utricular afferents carrying ipsilateral, caudal head tilt information onto medial canal afferents which carry ipsiversive head rotation information (Figure 6).

Axo-axonal connections between vestibular afferents have not previously been described, to the best of our knowledge. In spinal cord, primary afferents are well-known to receive axo-axonic inhibition, but this derives from central sources rather than from other afferents. As the data here are necessarily a static snapshot of one point in development, it is unclear whether these connections are stable. Both the activity evoked by synaptic transmission and neuronal transmitters themselves can serve as guidance cues (43). In addition, target-derived cues from the postsynaptic neuron (in this case medial canal afferents) might signal retrogradely (44), leaving open the possibility that canal afferents are instructor rather than student. Finally, it is also possible that these are simply ectopic, mistakenly formed synapses from initial exuberant connections that are destined for pruning, with no functional import. Selective expression of glutamate sensors in canal afferents would help to discern whether these connections are functional during development.

### Patterns of development

Though the serial-section EM dataset here is from a single timepoint in development, it is nonetheless helpful in evaluating developmental sequence in the vestibular system. Early-born neurons are thought to be likely to be myelinated first as well, because myelination is responsive to neuronal activity (45–47). Therefore, both the afferents and the central neurons that are already myelinated at 5.5 dpf are likely to have developed earlier than unmyelinated neurons, although not all central neurons will eventually be myelinated. From the standpoint of behavior, then, it makes sense that tangential and SVN neurons governing vestibulo-ocular reflexes are already myelinated, as are vestibulospinal neurons important in postural control (19). The commissural neurons described here (Figure 2) are also myelinated at this age, although myelination often dwindles after crossing the midline, suggesting that these are still in development. The remaining neurons described here with unidentified projections are likely to be even later-born (Figures 3, 4). Perhaps in reflection of this developmental gradient, 28/31 (90.3%) of identified commissural neurons received inputs from at least one myelinated utricular afferent. In contrast, only 28/46 (60.9%) of dorsal unknown neurons (Figure 3) and 43/83 (51.8%) of ventral unknown neurons (Figure 4) received input from at least one myelinated utricular afferent. We suggest that these differences indicate that earlier-born central neurons, as identified by myelinated axons at 5.5 dpf, tend to get input from earlier-born afferents that are also already myelinated at this developmental stage [see also (47)]. Thus, there may be a general pattern of wiring from early-born to early-born and late-born to late-born in the vestibular sensory system, similar to the pattern in motor systems (48–51).

Furthermore, there is a relatively low number of central neurons with predicted encoding of ipsilateral head tilt in the caudal-most (nose-up) direction (dark orange; see Figures 3, 4, compared with hair cells in Figure 1). None of the myelinated utricular afferents arise from the caudal zone, suggesting that pathways encoding caudal head tilt develop somewhat later than those encoding rostral head tilt. This feature would make sense in light of the observation that larval zebrafish at these ages are head-heavy, and tend to pitch forwards more than back (7). As a consequence, the pathways driving oculomotor and postural responses to nose-down pitch may need to develop earlier than those driving responses to nose-up pitch.

## Data availability statement

The datasets presented in this study can be found in online repositories. The name of the repository and accession number can be found below: CATMAID, https://zf.hms.harvard.edu/hildebrand16/data/vestibular_right.

## Ethics statement

The animal study was reviewed and approved by Washington University Animal Care and Use Committee.

## Author contributions

YJ and MWB carried out reconstructions and analysis. MWB prepared the figures and wrote the manuscript draft. Both authors revised and approved the submitted manuscript.

## Funding

This work was funded by NIH R01 DC016413 and R56 DC016413 (MWB). MWB is also supported by the Pew Scholar and McKnight Scholar Awards.

## Conflict of interest

The authors declare that the research was conducted in the absence of any commercial or financial relationships that could be construed as a potential conflict of interest.

## Publisher's note

All claims expressed in this article are solely those of the authors and do not necessarily represent those of their affiliated organizations, or those of the publisher, the editors and the reviewers. Any product that may be evaluated in this article, or claim that may be made by its manufacturer, is not guaranteed or endorsed by the publisher.
